# Protocol for immunodetection of α-synuclein pathology in paraffin-embedded liver tissues from murine models of Parkinson’s disease

**DOI:** 10.1016/j.xpro.2025.104158

**Published:** 2025-10-24

**Authors:** Martin Hallbeck, Maria Ntzouni, Martin Ingelsson, Juan F. Reyes

**Affiliations:** 1 Department of Biomedical and Clinical Sciences (BKV), Division of Cell Biology, Linköping University, Linköping, Sweden; 2 Linköping University Hospital Region Östergötland, 581 83 Linköping, Sweden; 3 Core Facility, Faculty of Medicine and Health Sciences, Linköping University, 581 83 Linköping, Sweden; 4 Krembil Brain Institute, University Health Network, Toronto, ON M5G 1P5, Canada; 5 Tanz Centre for Research in Neurodegenerative Diseases, Departments of Medicine and Laboratory Medicine & Pathobiology, University of Toronto, Toronto, ON M5G 1P5, Canada; 6 Department of Public Health and Caring Sciences, Molecular Geriatrics, Rudbeck Laboratory, Uppsala University, 752 37 Uppsala, Sweden

**Keywords:** Cell Biology, Molecular Biology, Neuroscience

## Abstract

The accumulation of α-synuclein (α-Syn) pathology in peripheral tissues of Parkinson’s disease (PD) has attracted growing scientific interest in recent years. Here, we present a protocol for the immunodetection of α-Syn pathology in murine liver tissue from models of PD using fluorescence microscopy. We describe steps for liver isolation, fixation, embedding, and immunodetection using an array of antibodies targeting α-Syn. This approach offers valuable applications for studying PD in transgenic mouse models and could be adapted for human liver tissue.

For complete details on the use and execution of this protocol, please refer to Hallbeck et al.^1^

## Highlights

• Direct protocol for detecting α-Syn pathology in paraffin-embedded murine liver tissue
• Integrates precise tissue handling, fixation, staining, and imaging for α-Syn detection
• Enables detection of total and modified α-Syn using antibody-based staining approaches
• Facilitates investigation of liver’s role in α-Syn clearance in Parkinson’s disease

## Abstract (editor highlights)

Publisher’s note: Undertaking any experimental protocol requires adherence to local institutional guidelines for laboratory safety and ethics.

## Graphical abstract

**Figure:**
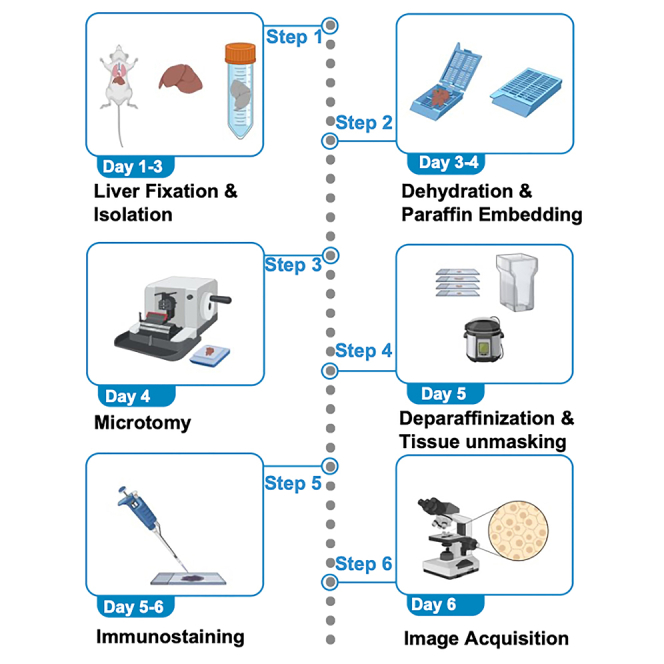

## Before you begin

In Parkinson’s disease (PD), alpha-Synuclein (α-Syn) pathology is not limited to the brain, as it has also been identified in multiple organs within the peripheral nervous system (PNS),^1^ and reviewed extensively.^2^^,^^3^ Thus, detection of α-Syn pathology in peripheral tissues may represent an early feature of PD pathogenesis^1^^,^^4^^,^^5^^,^^6^ and could potentially serve as a biomarker for disease diagnosis and progression.^2^^,^^3^ However, protocols for the immunodetection of α-Syn in peripheral tissues, whether from PD cases or in animal models of the disease, require reliable techniques to provide consistent results. The protocol herein offers a comprehensive method for the immunodetection of total and modified α-Syn pathology in paraffin-embedded murine liver tissue sections. We recently adopted this protocol for the immunodetection of total and modified α-Syn by tyrosine nitration (nY39), phosphorylation (pY39, pS87 and pS129, Y133) and C-terminal truncation events (X-122) in mouse models of PD (A30P, L61)^7^^,^^8^ and multiple system atrophy (MSA29).^9^ This protocol adds solutions to troubleshooting steps for tissue isolation, fixation and embedding, immunostaining and image analysis, helping to validate the presence of human α-Syn pathology in the liver, where we hypothesize its involvement in the clearance of PD associated pathology.

### Innovation

Detection of α-Syn pathology in peripheral tissues is an emerging and challenging area in Parkinson’s disease (PD) research. Existing immunohistochemical protocols are typically optimized for brain tissue and often do not translate well to peripheral organs such as the liver, where high autofluorescence, endogenous immunoglobulins, and variable antigen preservation can compromise reproducibility. This protocol provides a fully integrated, step-by-step workflow spanning perfusion, liver isolation, fixation, embedding, automated sectioning integrated with a waterflow system, epitope retrieval, multiplex fluorescence immunostaining, and imaging that is specifically optimized for paraffin-embedded murine liver tissue. Such key innovations include: Multiplex detection of total and post-translationally modified α-Syn species (e.g., phosphorylation, truncation) within the same tissue section, enabling detailed molecular profiling. Troubleshooting strategies to overcome liver-specific challenges, such as the use of mouse-on-mouse blocking, lipofuscin quenching, and antibody titration adjustments for different antibody clones and batches. Flexibility and adaptability, allowing application to multiple PD and MSA mouse models and to human liver tissue, and compatibility with other antibodies or molecular probes for investigating related pathologies (e.g., inflammation or aggregate co-localization).

By combining optimized tissue processing with tailored immunostaining strategies, this protocol achieves a high signal-to-noise ratio and consistent detection of α-Syn pathology in a peripheral organ. Thus, it is opening a new avenue for studying α-Syn distribution beyond the central nervous system and for assessing potential peripheral mechanisms of PD progression and clearance.

### Institutional permissions

This protocol requires tissue from murine models and experiments must be conducted under ethical approvals by internal animal welfare institutions and national authorities responsible for animal experimentation before starting these procedures. All experiments described in this protocol have been approved by the Animal Experimentation Ethics Committee at Linköping University and the Swedish Board of Agriculture (Dnr 8142-2021).

## Key resources table

**Table:** 

REAGENT or RESOURCE	SOURCE	IDENTIFIER
**Antibodies**		

Syn211 (0.3 μg/mL)	Abcam	Syn211, RRID:AB_1810987
4B12 (0.3 μg/mL)	GeneTex	4B12, RRID:AB_380314
MJFR 14-6-2 (1.5 μg/mL)	Abcam	AB209538, RRID:AB_N/A
IgG goat anti-mouse Alexa Fluor 488 (4 μg/mL)	Life Technologies	Cat#A11029:RRID:AB_2534088
IgG goat anti-rabbit Alexa Fluor 564 (4 μg/mL)	Life Technologies	Cat#A11012: RRID:AB_2534079

**Chemicals, peptides, and recombinant proteins**		

50 mL syringe	Sigma-Aldrich	Cat#XX1105005
1× PBS pH 7.4	Gibco	P4417-50TAB
4% paraformaldehyde (PFA)	Santa Cruz Biotechnology	Cat#sc-281692
Ethanol 70%	SOLVECO, Rosersberg, Sweden	Cat# 1054
Ethanol 99.7%	SOLVECO, Rosersberg, Sweden	Cat#1125
HistoClear (xylene substitute)	Histolab Products AB, Askim, Sverige	Cat#14250
Paraffin Histowax	Histolab Products AB, Askim, Sverige	Cat#00402-1
Histology cassettes	Sigma-Aldrich	Cat#H0542-1CS
Metallic molds	MedSupply Partners (MSP)	30-M4743 medsupplypartners.com
Bovine serum albumin (BSA)	Sigma-Aldrich	Cat#A3912
Fetal bovine serum (FBS)	Life Technologies	Cat#A5256801
Xylene	Sigma-Aldrich	Cat#534056
Butterfly needle	B. Braun	Cat# 4056501-01
Sudan Black B	Sigma-Aldrich	Cat#199664
Triton X-100	Sigma-Aldrich	Cat#T8787
50 mL Falcon tubes	Sigma-Aldrich	Cat#CLS253070
Hoechst staining reagent	Invitrogen	Cat#H3570
Low pH epitope retrieval solution	Agilent Technologies	Cat#K8005
Whatman filter paper	Sigma-Aldrich	Cat#WHA1003185
Hydrophobic pen barrier	Thermo Fisher Scientific	Cat#R3777
Superfrost Plus glass slides	Thermo Fisher Scientific	Cat#J1800AMNZ
VECTASHIELD antifade mounting media	Vector Laboratories	Cat#H-1900

**Critical commercial assays**		

Mouse-on-mouse kit (M.O.M)	Vector Laboratories	Cat#FMK2201
Vector TrueVIEW solution	Vector Laboratories	Cat# SP8400-15

**Experimental models: Organisms/strains**		

Male (Thy-1)-h(A30P)-α-synuclein mouse line expressing human α-Syn with the A30P-mutation under the Thy-1 promoter aged 18 months was used.	Laboratory of Professor Philipp Khale	N/A

**Software and algorithms**		

ImageJ (Fiji)	–	https://imagej.net/ij/

**Other**		

PT Link, DAKO PT200	Agilent, DAKO	Cat#PT200 PT Link, Pre-Treatment Module for Tissue Specimens | Agilent
Tissue processor, Leica TP1020	Leica Biosystems	Leica TP1020 | Automatic Benchtop Tissue Processor
Embedding station, Leica EG 1150 H&C	Leica Biosystems	Leica EG1150 Modular Tissue Embedding Center | Leica Biosystems
Microtome, Microm HM 355S	Thermo Scientific	Rotary Microtome Microm HM 355S - 387861
LSM 700 confocal microscope	Zeiss	RRID:SCR_017377
Linear stainer, Leica ST4020	Leica Biosystems,	Leica ST4020 | Linear Slide Stainer, Compact

## Materials and equipment

### Triton permeabilization solution

• Prepare Triton-X-100 permeabilization buffer by adding 800 μL of Triton-X-100 solution into 200 mL 1× PBS (pH 7.4) solution.
• Mix with a magnetic stirrer until fully dissolved.
• Store at room temperature for 1 month (23°C).

**Table:** 

Reagent	Final concentration	Amount
1× PBS (pH 7.4)	N/A	199.2 mL
Triton-X-100	0.4%	800 μL
Total	N/A	200 mL

**CRITICAL:** Triton-X-100 is very viscous. To make this solution more efficiently, cut the tip of a 1 mL pipette tip with a razor blade to make a larger opening then proceed aspirating the Triton-X-100 slowly. Rinse the pipette 3 to 4 times into the solution to remove any remaining Triton-X-100 attached to the tip.

### Tissue blocking solution

• Prepare 10 grams of BSA into 100 mL of 1× PBS.
• Mix by vigorous vortexing until fully dissolved.
• Prepare daily as it is a source of bacterial growth.

**Table:** 

Reagent	Final concentration	Amount
BSA	10%	10 grams
1× PBS (pH 7.4)	N/A	100 mL
Total	N/A	100 mL

***Alternatives:*** The use of 5% fetal bovine serum (FBS) could be used as blocking solution, but caution must be taken as is prone to bacterial contamination.

### Antibody dilution buffer

• Prepare 100 milligrams of BSA into 10 mL of 1× PBS.
• Add 0.1 mL of Triton-X-100.
• Mix by vortexing and keep at 4°C.
• Prepare daily as it is a source of bacterial growth.

**Table:** 

Reagent	Final concentration	Amount
BSA	1%	100 mg
1× PBS (pH 7.4)	N/A	10 mL
Triton-X-100	0.1%	0.1 mL

### Lipofuscin blocking solution

• Prepare 100 milligrams of Sudan block B into 100 mL of 70% Ethanol.
• Mix with a magnetic stirrer for 1 h.
• Prepare daily as it is a source of bacterial growth.

**Table:** 

Reagent	Final concentration	Amount
Sudan Black B	0.1%	100 mg
Ethanol	99.7%	100 mL
Total	N/A	100 mL

**CRITICAL:** To clear all precipitate from the lipofuscin blocking solution slowly filter over a glass bottle by gravity using a Whatman filter paper (Repeat if necessary).

***Note:*** Tissue overstaining using Sudan Black B could be easily removed by dipping the slides in 70% ethanol 1–3 s.

## Step-by-step method details

### Tissue perfusion and isolation—Day 1–3

**Timing: 3 h**

The following steps enable the isolation of complete mouse livers while eliminating antibody cross-reactivity with endogenous immunoglobulins.

**Figure 1:**
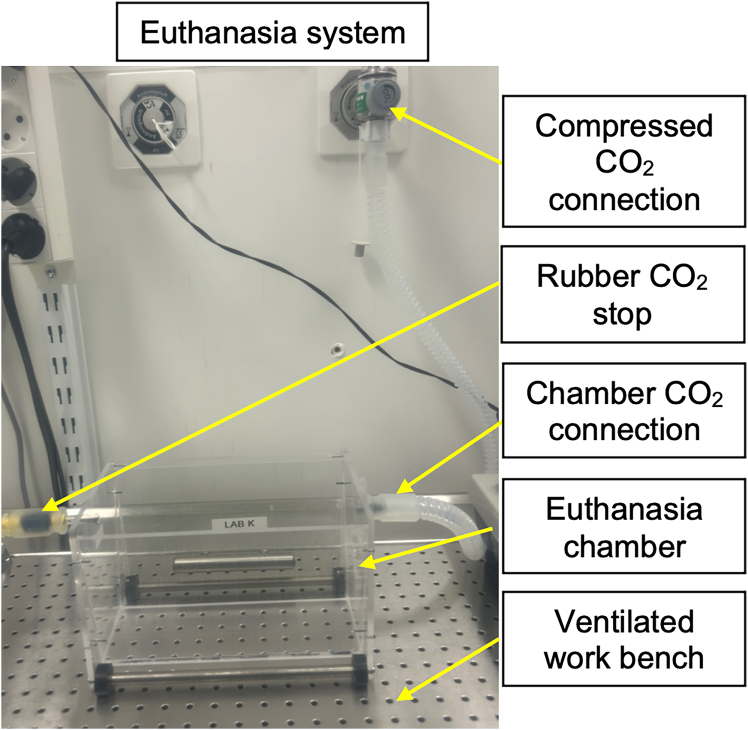
The mouse euthanasia system A clear euthanasia chamber connected to wall compressed CO_2_ with an exit blocked with a rubber stop placed over a ventilated bench.

1. Connect the CO_2_ to the euthanasia chamber and block the exit with a rubber stop, as shown in Figure 1.

**CRITICAL:** Ensure the euthanasia chamber is clear to visualize the mouse throughout the procedure and monitor its reaction to CO_2_.

***Note:*** This step uses compressed CO_2_ for euthanasia allowing direct delivery to the chamber with equipment suitable for small rodents (mice). Policies allowing for euthanasia vary by location, medical standards, and ethical considerations. Please refer to the ethical framework of your institution.

2. Place the mouse in the chamber and introduce CO_2_ gradually, increasing from 30% to 70% per minute over 2–3 min.
  a. Maintain CO_2_ exposure for an additional 60 seconds after visual confirmation of respiratory cessation to ensure complete euthanasia.
  b. Clean the euthanasia chamber between each use to remove odors or biological material from the previous session.
3. Remove the mouse from the chamber and confirm death by checking for fixed and dilated pupils.

**CRITICAL:** If the CO_2_ procedure does not work within the specified time, check for possible CO_2_ leakage or improper delivery to the chamber.

**Figure 2:**
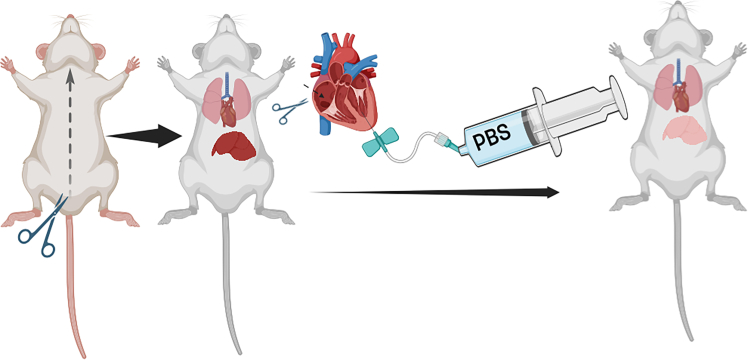
Schematic overview of major perfusion steps Schematic representation of a mouse with paws fixed to the bench showing the incision at the mid-belly to expose the thoracic cavity and internal organs followed by perfusion with PBS (Figure generated with BioRender).

4. Fix the paws to the laboratory bench using laboratory tape to secure the mouse for dissection.
5. Lift the skin at the mid-belly and make an incision extending to the chest cavity to reveal the thoracic cavity and internal organs (Figure 2).

**CRITICAL:** Cut the diaphragm immediately after revealing internal organs, as it serves as a secondary method of euthanasia.

***Note:*** Use thick scissors to cut the rib cage for easier access to the heart, improving perfusion efficiency.

6. Prepare a butterfly needle connected to a 50 ml syringe containing cold (4°C) 1× PBS solution (pH 7.4).
7. Once the heart is revealed insert the needle through the left ventricle (halfway through the heart).
  a. Make a small incision in the right atrium.
  b. Gently inject at least 30 mL of PBS to flush out all the blood until the liver becomes light pink, ensuring complete blood removal (troubleshooting: problem 1).

***Note:*** If the tissue is not required for other applications where proteins cannot be cross-linked such as Western blot or ELISA (Enzyme linked immunosorbent assay), it is advisable to perform perfusion with at least 30 mL of cold (4°C) 4% paraformaldehyde (PFA) immediately following the saline solution to improve tissue fixation.

8. Locate and isolate the liver by moving the intestines to the side using a small spatula,
  a. Use blunt forceps and a sharp pair of small scissors to cut the portal vein and vena cava to detach the liver without poking or damaging the lobes.
9. Immerse the entire or part of the liver (as needed) in cold (4°C) 4% PFA (20 mL/liver) in a 50 ml Falcon tube at 4°C for 48 h.

***Note:*** Step 9 is necessary even if perfusion with 4% PFA is performed as it ensures proper tissue fixation.

**CRITICAL:** Do not keep livers in PFA solution at room temperature (23°C) during the experimental process as this increases oxidation and reduces fixation effectiveness. Incubate the solution on wet ice during the entire process.

### Tissue dehydration and clearing—Day 3–4

**Timing: 12 h (overnight)**

The following steps enable dehydration and paraffin infiltration to tissues using an automated tissue processor.

10. Incubate the whole or part of the liver in 1× PBS (pH 7.4) for 15 min before proceeding with the dehydration process.
11. Transfer specimens from tubes into labeled histology cassettes and immerse them in 70% ethanol.

***Note:*** Label cassettes using a pencil, as permanent markers or sharpies fade rapidly in ethanol.

12. Place the cassettes in the automatic tissue processor (Figure 3).
  a. Immerse sections in a series of alcohol baths with increasing ethanol solutions for dehydration, HistoClear solution for clearing, and paraffin baths for paraffin infiltration (Table 1).

**Table 1:** Tissue processor dehydration & paraffin infiltration protocol

Step	Chemicals	Vacuum/Temp	Volume	Time
1	70% ethanol	V/ RT	1.8 L	0.5 h
2	90% ethanol	V/ RT	1.8 L	1 h
3	95% ethanol	V/ RT	1.8 L	1 h
4	95% ethanol	V/ RT	1.8 L	1 h
5	99.7% ethanol	V/ RT	1.8 L	0.5 h
6	99.7% ethanol	V/ RT	1.8 L	0.5 h
7	99.7% ethanol	V/ RT	1.8 L	1 h
8	HistoClear	V/ RT	1.8 L	1 h
9	HistoClear	V/ RT	1.8 L	1 h
10	HistoClear	V/ RT	1.8 L	1.5 h
11	Paraffin	V/ 59°C	1.8 L	1.5 h
12	Paraffin	V/ 59°C	1.8 L	1.5 h
Total time	N/A	N/A	N/A	12 h

**Figure 3:**
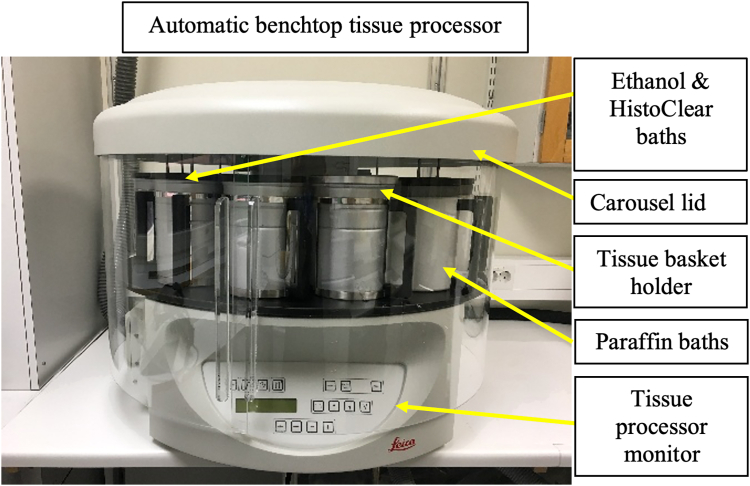
Automatic benchtop tissue processor (Leica TP1020)
13. After the protocol is completed, immediately transfer the tissue cassettes to the hot storage shelves of the embedding station (Figure 4).

**Figure 4:**
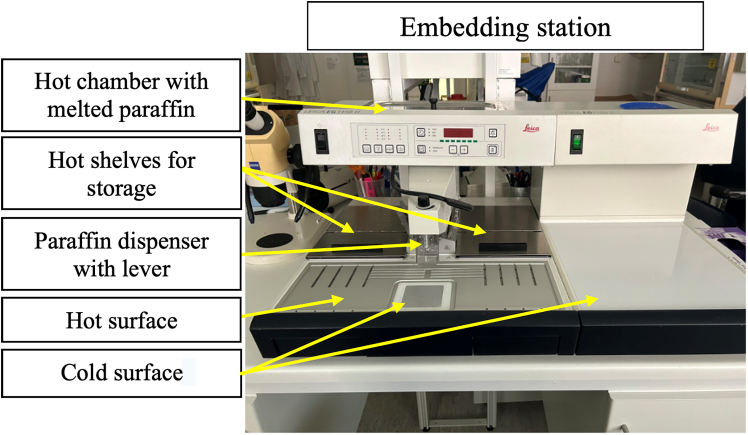
Components of the embedding station Embedding station with the hot plate on the left and cold plate on the right Note that the metal molds are placed within the chambers covered by black lids (Leica EG1150 Modular Tissue Embedding Station).

***Note:*** The hot embedding station includes a heated chamber, a hot metal surface, two small warm storage compartments, and a hot dispenser which releases melted paraffin, as well as a cold metal area that solidifies the paraffin quickly, enabling proper tissue orientation for sectioning (Figure 4).

**CRITICAL:** Set the starting embedding time at the hot part of the embedding station the day before to ensure the paraffin is hot (60°C) on the embedding time (paraffin needs at least 2 h to melt). The cold plate of the embedding station reaches the appropriate temperature (−20°C) and solidifies the wax within a few minutes.

### Paraffin embedding—Day 4

**Timing: 5 min/sample**

The following steps allow for preparation of tissue for embedding and orientation on slides.

14. Place the metallic molds (suitable for the size of your tissue) on the hot surface of the metalic plate (60°C) and allow them to adjust to temperature before use.

***Alternatives:*** Plastic disposable transparent molds are also commercially available and also suitable for paraffin embedding of liver tissues.

15. Transfer the histology cassettes from the storage hot shelves to the hot surface of the embedding station (Figure 4).

**CRITICAL:** Avoid skin contact with melted paraffin as it may cause a slight burn. Always use long blunt forceps to handle tissue. The hot metal molds could be handled by hand (with gloves) but if sensitivity to heat is high the use of forceps is recommended.

**Figure 5:**
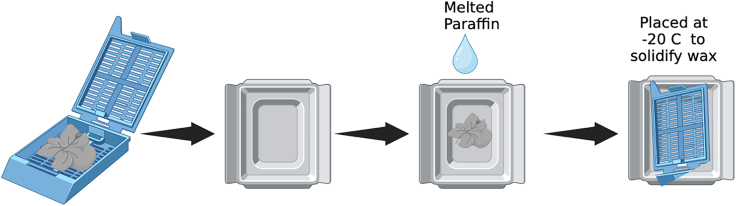
Overview of the embedding process Schematic representation of liver placement into the metal mold, the addition of melted paraffin, covering the mold with the cassette lid, and placement on the −20°C embedding surface to allow for solidification (Figure generated with BioRender).

16. Take the cassette with warm forceps and position the liver tissue in the center of the metallic mold and dispense melted paraffin by pressing the lever on the embedding station (Figure 5, Methods video S1).

***Note:*** Place the tissue in the middle of the metallic mold with the large lobes facing down as this is the area that will be sectioned first (Figure 5).

17. Fill the mold completely with melted wax until the specimen is fully covered (Methods video S1).
18. Place the cassette with the label on top and allow it to solidify along with the sample on the cold station (around 15 min) (Methods video S1).
19. After the wax is solidified detach the metallic mold from the paraffin embedded samples and proceed to the next step for sectioning.

***Note:*** The liver cassettes could be stored at room temperature (23°C), for long periods of time but should be placed at 4°C overnight (18–24 h)**,** or at −20°C for 30 min prior to sectioning. The cooling system within the microtome helps keeps the cassette at a cool temperature during the sectioning process.

Methods video S1. Paraffin embedding liver tissue sections (paraffin embedding), related to step 16

### Paraffin sectioning—Day 4

**Timing: 3 min/section**

The following steps enable sectioning of liver tissue with proper orientation on glass microscopy slides.

**Figure 6:**
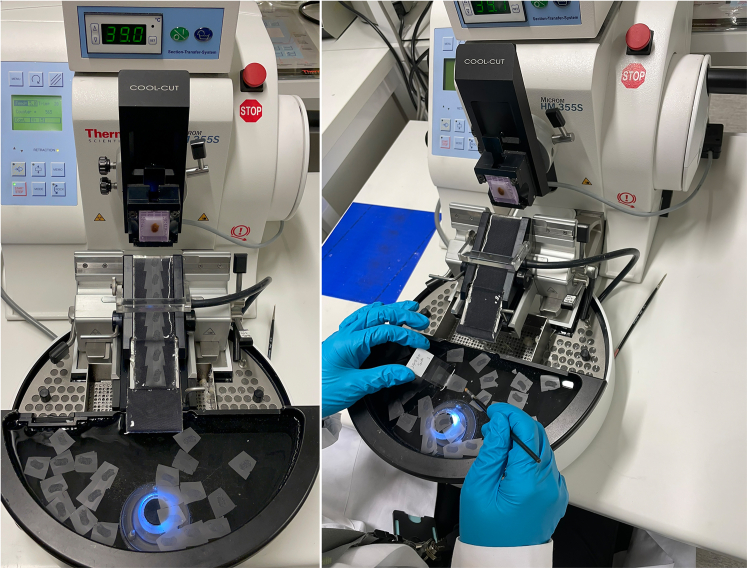
Sectioning station Side and front views of the Microm HM 355S rotary automated paraffin microtome, featuring an integrated cool-cut sample paraffin block holder, a transfer water flow system, and a warm bath with sections floating on its surface.

***Note:*** Trim the paraffin around the specimen using a razor blade to eliminate unnecessary wax surrounding the tissue which increases the area and reduces the number of sections per slide. While trimming, it is helpful to make a pyramid/triangular shape so that the orientation can be distinguished when multiple sections are placed on the glass slide. This is especially important because the sections are automatically transferred to the warm water bath by the microtome’s integrated water flow system, where they often rotate. The unique wax shape serves as a reliable marker to identify tissue orientation (Figure 6, Methods video S2).

20. Place the cassette containing the paraffin block on the cold sample holder (cool-cut) of the automated paraffin microtome (Figure 6, Methods video S2).
21. Set the feed to 5 μm and sectioning speed to 15 mm/s and start the automated sectioning (Methods video S2).

22. Allow the sections to slide through the water flow and let them stretch on the warm water bath (39°C) for 1–2 min, this removes any wrinkles the tissue acquires during sectioning (Methods video S2).

**CRITICAL:** Avoid keeping the tissue for long periods of time in the warm water bath, as this causes sections to tear.

23. Transfer the desired number of sections onto commercially available Super-Frost Plus adhesive glass microscopy slides using small painting brushes.
  a. Place the glass slide beneath the sections and gently lift the slide as soon as one side of a section touches the glass.
24. Dry the sections overnight (24 h) at room temperature (23°C), with proper room ventilation.

***Note:*** If slides are placed on a slide box, keep the box lid open to ensure tissue dryness.

**Pause point:** If necessary, sections could be stored for a few weeks at room temperature (23°C)**,** before continuing the deparaffinization and immunostaining steps.

**CRITICAL:** Use fresh-cut sections whenever possible as prolonged storage may lead to epitope reduction.

Methods video S2. Microtome sectioning of liver tissue sections (tissue sectioning), related to step 20

### Deparaffinization and epitope retrieval—Day 5

**Timing: 2.5 h**

The following immunostaining solutions are water-soluble thus deparaffinization of tissue sections is necessary before proceeding with the immunostaining protocol.

**Figure 7:**
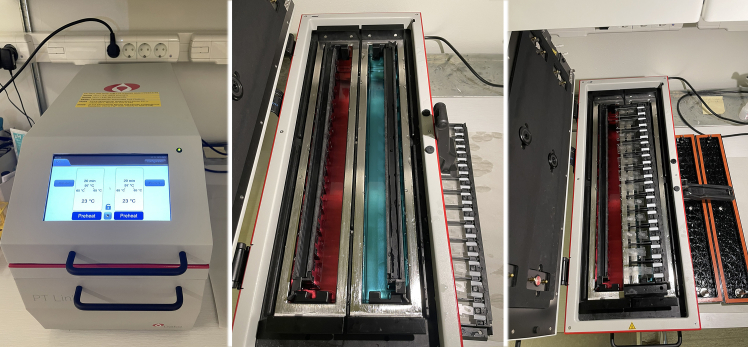
Antigen retireval system Closed and open side views of the automatic epitope retrieval system (PT Link, pre-Treatment module for tissue specimens).

25. Fully immerse the desired number of slides in xylene solution three times, for 5 min each.
26. Transfer slides to containers with decreasing ethanol concentrations three times each for 5 min in the following order.
  a. 100% ethanol.
  b. 70% ethanol.
  c. 50% ethanol.
  d. Deionized water.
27. Place sections into a PT Link epitope retrieval system using the commercially available low pH solution (pH 6.1) following the manufacturer’s protocol (Figure 7, https://www.agilent.com/store/productDetail.jsp?catalogId=K800521-2).
  a. Pre-heating (to 65°C): 25 min.
  b. Heating (up to 97°C): 20 min.
  c. Cooling down (to 65°C): 34 min.

**CRITICAL:** Some antibody epitopes may be enhanced or abolished using this technique, skip this step if epitope retrieval diminishes the desired epitope (troubleshooting: problem 2).

28. Transfer slides to 1× PBS (pH 7.4) for 10 min to remove excess solution from the previous step.

**CRITICAL:** Perform immunostaining on tissue the same day for optimal results. If immediate processing is not possible, store slides at 4°C until next day.

### Immunostaining of liver tissue sections for fluorescence microscopy—Day 5–6

The following steps enable the fluorescence detection of α-Syn in liver tissue sections from murine models of PD.

#### Immunostaining protocol day 5

**Timing: 4 h**

29. Incubate tissue slides in Triton Permeabilization Solution for 45 min at room temperature (23°C), with gentle shaking on a bench-top shaker.
30. Rinse slides with 1× PBS (pH 7.4) for five minutes to remove excess Triton-X-100 and repeat this step twice for a total of 15 min.
31. Block slides in Tissue Blocking Solution for 1 h at room temperature (23°C), with gentle shaking on a bench-top shaker (troubleshooting: problem 3).

***Note:*** When too much background is observed using primary antibodies, commercially available kits can substantially help reduce non-specific background fluorescence such as the mouse-on-mouse (M.O.M.) fluorescence blocking kit or the autofluorescence quenching kit (Vector Labs).

32. Quickly dry slides around the tissue using a napkin with care not to scratch the tissue.
33. Create a hydrophobic barrier around the tissue using a hydrophobic pen as this step significantly decreases the amount of antibody used.

**CRITICAL:** Ensure that tissue on the slides do not dry out while creating the barrier as this will increase non-specific staining.

34. Incubate slides in primary antibody in with Antibody Dilution Buffer and incubate overnight (18–24 h) at 4°C.

**CRITICAL:** Sections where primary and secondary have been omitted should be included in the experimental set up as they are helpful to assess the degree of fluorescence background within the tissue.

***Note:*** Two primary antibodies could be added in this step if they are generated from different species and preferably, with no overlapping epitopes to avoid competitive binding.^1^ Titration of antibodies are necessary by experimenter as slight batch to batch variations and concentration of antibody may affect tissue staining (troubleshootingproblem 4).

#### Immunostaining protocol—Day 6

**Timing: 3.5 h**

35. Next day, incubate the entire slide in 1× PBS (pH 7.4) for 5 min with gentle shaking on a bench-top shaker, repeat twice for a total of 15 min.
36. Incubate tissue (area under the hydrophobic barrier) in secondary antibody prepared in antibody dilution buffer with the desired fluorescence marker (e.g., Alexa 488 goat anti-rabbit) for 2 h at room temperature (23°C) and cover with foil to prevent photobleaching.
37. Incubate the entire slide slides 1× PBS for 5 min with gentle shaking on a bench-top shaker, repeat twice for a total of 15 min.

***Note:*** If nuclear staining is desired, incubate the area under the hydrophobic barrier in Hoechst solution for 10 min then rinse entire slide with 1× PBS for additional 10 min.

38. Incubate slides in lipofuscin blocking solution for 1–3 min to eliminate endogenous lipofuscin background, followed by a quick dip (1 sec.) in 70% ethanol to remove unbound material (troubleshooting: problem 1).
39. Rinse slides in 1× PBS (pH 7.4) for 10 min.
40. Add 1–2 drops (depending on tissue size) of Vectashield fluorescence mounting media and cover slip immediately.
41. Remove excess media by gently pressing on the coverslip using a dry paper towel, this also removes any potential bubbles generated following addition of the mounting media.
42. Seal the coverslip and glass slide using standard nail polish solution and allow it to fully dry (approximately 30 to 60 min) before using the confocal microscope.

***Note:*** Immunostained slides should be protected from light while the nail polish is drying to prevent photobleaching.

### Image acquisition and confocal microscopy—Day 6

**Timing: 0.5–1 h (per section)**

43. Gently wipe the slides with a damp (70% ethanol) microscope tissue ensuring not to break the nail polish seal which will cause the cover slip to move preventing a clear focus.
44. Start the confocal microscope and acquire images from control sections where primary and secondary antibodies have been omitted to set the microscope with appropriate laser settings.

***Note:*** This step allows you to identify how much background fluorescence is present in your liver tissue sections.

45. Clean slides gently with 70% ethanol ensuring not to break the nail polish seal. Once the settings have been acquired, analyze liver tissue from experimental conditions with the same settings generated from the controls.

***Note:*** Images can be taken with the 20× objective without oil but if higher resolution is needed, the 40× objective can be used using microscopy oil.

46. Semi-quantitative assessment could be performed when comparing different animal models, ages or different treatments using the overall number of inclusions per section and graded as +, ++, +++ (see Hallbeck et al., 2024,^1^ troubleshooting: problem 5).
47. After images have been acquired, store tissues at 4°C and protect from light if additional imaging is later required.

***Note:*** Fluorescence slightly degrades over time but may be stable for at most 2-weeks. However, imaging of recently immunolabeled tissue is highly recommended.

**CRITICAL:** Once the microscope settings have been identified, it is important to keep the settings the same throughout the experiments if comparisons are made using fluorescent intensity in different tissues.

## Expected outcomes

**Figure 8:**
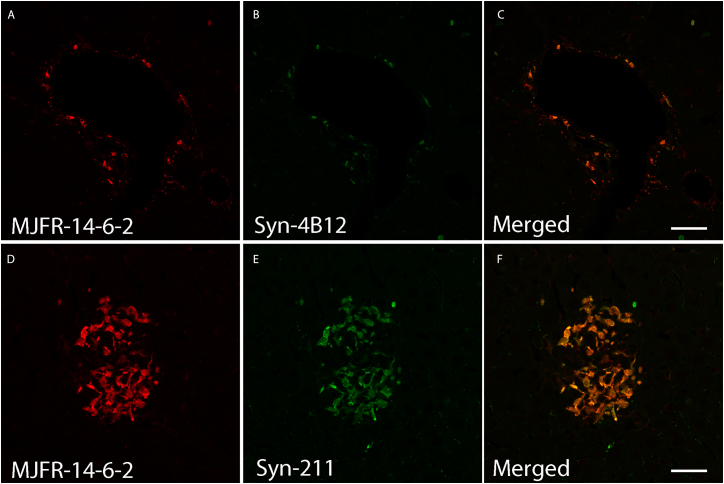
Immunodetection of α-Syn pathology within the liver of the A30P mouse model of PD Liver tissue sections from the A30P mouse model of PD showing human α-Synuclein inclusions labeled with the aggregate-specific α-Syn antibody MJFR-14-6-2 and Syn-4B12 (panels A–C), and MJFR-14-6-2 and Syn-211 (panels D–F). Scale bars = 50 μm.

After imaging, the presence of α-Syn pathology within the liver in transgenic models of PD should be readily visible as shown in Figure 8.^1^ In some instances, the identity of the α-Syn inclusions may be punctate or diffuse a process which is highly dependent on the age and/or the transgenic model used (see limitations). For instance, diffuse inclusions and small punctate are observed in the A30P, L61 and MS29 mouse models,^1^^,^^6^ whereas small punctate are observed in non-transgenic mice following seeded with recombinant mouse preformed fibrils (PFFs). Moreover, the presence of α-Syn pathology in the liver may vary depending on the antibody used (modified vs total α-Syn) or the animal model used (see the limitations section below). In our hands the C-terminal antibodies (MJFR-14-6-2, 4B12, 14H, 211, 202) label very clear α-Syn pathology whereas the N-terminal antibodies appear less reactive (PA1, PA5) unless epitopes are modified such as nY39, pY39.^1^

## Limitations

Several animal models of PD have been generated to express human α-Syn to different degrees in the brain depending on the promoter used and/or the transcripts expressed.^6^ We have observed different degrees of pathology in transgenic mice, with the A30P model showing the most pathology, followed by the L61 model and the MSA-29 model (for multiple system atrophy). Thus, based on the expression and deposition of α-Syn in the brain, the amount of α-Syn pathology in the liver could fluctuate depending on the animal model used. Additionally, whether the model was seeded with fibrillar assemblies (PFFs) to accelerate α-Syn aggregation either from the brain or in peripheral tissues could also affect the amount of α-Syn pathology present in the liver. In this scenario, it is difficult to identify exogenous vs endogenous assemblies as antibodies will detect both proteins. In some instances, however, it may be possible to differentiate between the seeding material from the endogenous expression of α-Syn by using truncated PFFs which lack the C-terminal domain (1-120) thus preventing the use of C-terminal antibodies which epitopes rely on this region including the widely used pS129 antibodies which target phosphorylated α-Syn at position 129, see Hallbeck et al., 2024.^1^

## Troubleshooting

### Problem 1

Lack of proper perfusion (Tissue perfusion steps).

### Potential solution

• If the liver does not turn pale pink after perfusion with PBS solution or 4% PFA, this may be due to a problem with the perfusion technique. This will result in the presence of endogenous antibodies present in the blood. We suggest the use of the M.O.M. kit described above to eliminate endogenous immunoglobulins. Additionally, the use of sections with and without antibodies (both primary and secondary) should be used to assess the levels of endogenous immunoglobulins (background fluorescence) and whether true signal could be differentiated from endogenous immunoglobulin signal.

### Problem 2

Epitope Retrieval (Immunostaining of liver tissue steps).

### Potential solution

• It is highly recommended that non-epitope retrieved sections and those subjected to epitope retrieval using adjacent sections are compared for each antibody tested, given that the near boiling temperature of the PT-Link system during epitope retrieval (95°C) may abolish certain epitopes.
• We have noticed that most antibodies targeting α-Syn slightly enhanced antibody staining compared to sections that did not undergo this process. However, when using probes targeting total aggregated proteins (non-synuclein specific), such as oligothiophenes (LCO's),^6^ a slight decrease in staining was observed. Thus, we hypothesize that the use of high temperatures for long periods of time using this system may decrease protein aggregate staining, pointing to the necessity of testing this technique for α-Syn antibodies not shown in this protocol.

### Problem 3

Endogenous immunoglobulin detection (Immunostaining of liver tissue steps).

### Potential solution

• Too much background autofluorescence resulting from endogenous immunoglobulins often arises following partial liver perfusion, but this problem can be significantly reduced using commercially available kits that substantially reduce endogenous immunoglobulins such as mouse-on-mouse kit (M.O.M, Vector Labs, Cat#FMK2201) following the manufacturer’s instructions (https://vectorlabs.com/productattachments/protocol/VL_FMK-2201_UserGuide_LBL02255.pdf).
• Alternatively, if the autofluorescence results from lipofuscin which increases with aging, Sudan Black B can be used as stated in this protocol.
• On the other hand, autofluorescence resulting from non-lipofuscin sources can be reduced using a quenching kit which diminishes autofluorescence (Vector True View, Vector Labs, Cat# SP8400) as per manufacturer’s instructions (https://vectorlabs.com/productattachments/protocol/VL_SP-8400_UserGuide_LBL02293.pdf).

### Problem 4

Antibody Titration (Immunostaining of liver tissue steps).

### Potential solution

• All antibodies used for α-Syn staining used here and in our previous manuscript are commercially available through different companies. Although the same antibody clone is provided, we found variability in the intensity of the staining when different batches were used, and this effect was at times due to the different concentrations in which the antibody was provided (1 mg/mL vs 0.5 mg/mL). Thus, caution must be taken when titrating antibodies from different batches ensuring that the same final concentration is used.

### Problem 5

Semi-quantitative analysis (Image acquisition and confocal microscopy).

### Potential solution

• Semi-quantitative analysis of α-Syn pathology could estimate the relative abundance without providing exact numerical values. This is a common practice when exact values are difficult to obtain. When performing semiquantitative analysis of pathology in the liver tissue it is recommended to do the following:
  ○ Perform the analysis on multiple biological replicates (a cohort) containing suitable controls.
  ○ Repeat the scoring or measurement to assess consistency or have trained observers (colleagues) score the same data independently.
  ○ If possible, compare semi-quantitative results with a fully quantitative techniques (if available such as Western blot or ELISA.

## Resource availability

### Lead contact

Further information and request for resources and reagents should be directed to and will be fulfilled by Juan F. Reyes (juan.reyes@liu.se).

### Technical contact

Further information and questions about the technical specifics of performing the protocols should be directed to Juan F. Reyes (juan.reyes@liu.se).

### Materials availability

This study did not generate new reagents.

### Data code availability

This study did not analyzed/generated data sets/codes.

## Acknowledgments

We thank Dr. Vesa Loitto from the core imaging facility at Linköping University for technical assistance. The authors gratefully acknowledge the financial support from the 10.13039/501100001862Swedish Research Council (2019-01016), the 10.13039/501100007416Swedish Brain Foundation, The Östergötland Research Foundation for Parkinson’s Disease, The Swedish Dementia Foundation, The Swedish Parkinson’s Foundation, The 10.13039/501100005701Åhlén Foundation, and 10.13039/100007435Åke Wibergs Stiftelse. Funding agencies were not involved in the design or interpretation of the study.

## Author contributions

J.F.R. and M.H. conceived and performed the study. J.F.R. and M.N. wrote the original article. M.N. performed tissue dehydration and clearing, paraffin embedding, and tissue sectioning. J.F.R. performed immunodetection and imaging. M.H., M.N., M.I., and J.F.R. discussed the results and edited the article. All authors were involved in the discussion of results and edited the final version of the article.

## Declaration of interests

M.I. is a paid consultant to BioArctic AB and to Eisai Pharmaceuticals.
